# Non-invasive enhancement of intracortical solute clearance using transcranial focused ultrasound

**DOI:** 10.1038/s41598-023-39640-2

**Published:** 2023-07-31

**Authors:** Seung-Schik Yoo, Evgenii Kim, Kavin Kowsari, Jared Van Reet, Hyun-Chul Kim, Kyungho Yoon

**Affiliations:** 1 Department of Radiology, Brigham and Women’s Hospital, Harvard Medical School, 75 Francis Street, MA 02115 Boston, USA; 2 Department of Artificial Intelligence, Kyungpook National University, Daegu, Republic of Korea; 3 School of Computational Science and Engineering, Yonsei University, Seoul, Republic of Korea

**Keywords:** Biotechnology, Neuroscience

## Abstract

Transport of interstitial fluid and solutes plays a critical role in clearing metabolic waste from the brain. Transcranial application of focused ultrasound (FUS) has been shown to promote localized cerebrospinal fluid solute uptake into the brain parenchyma; however, its effects on the transport and clearance of interstitial solutes remain unknown. We demonstrate that pulsed application of low-intensity FUS to the rat brain enhances the transport of intracortically injected fluorescent tracers (ovalbumin and high molecular-weight dextran), yielding greater parenchymal tracer volume distribution compared to the unsonicated control group (ovalbumin by 40.1% and dextran by 34.6%). Furthermore, FUS promoted the drainage of injected interstitial ovalbumin to both superficial and deep cervical lymph nodes (cLNs) ipsilateral to sonication, with 78.3% higher drainage observed in the superficial cLNs compared to the non-sonicated hemisphere. The application of FUS increased the level of solute transport visible from the dorsal brain surface, with ~ 43% greater area and ~ 19% higher fluorescence intensity than the unsonicated group, especially in the pial surface ipsilateral to sonication. The sonication did not elicit tissue-level neuronal excitation, measured by an electroencephalogram, nor did it alter the molecular weight of the tracers. These findings suggest that nonthermal transcranial FUS can enhance advective transport of interstitial solutes and their subsequent removal in a completely non-invasive fashion, offering its potential non-pharmacological utility in facilitating clearance of waste from the brain.

## Introduction

The lymphatic system plays a major role in the transport/removal of metabolic byproducts and waste from the body, which are collected by networks of lymphatic vessels widely distributed throughout the organs. The central nervous system (CNS), however, lacks dedicated lymphatic vessels within neuronal parenchyma while the relatively high metabolic rate of neuronal cells and their high susceptibility toward changes in the extracellular environment necessitate efficient waste clearance for normal function. Studies have reported associations between aberrant brain waste clearance with dementia and Alzheimer’s disease (AD)^1,2^ as well as with aging^3,4^. On this basis, the mechanisms of lymphatic function of the CNS have gathered significant research interest.

The brain and the spinal cord are immersed in cerebrospinal fluid (CSF), which is primarily produced by the choroid plexus that lines cerebral ventricles^5^. The space between neuronal cells including the extracellular matrix (*i.e.,* the interstitial space) contains interstitial fluid (ISF), which is compositionally similar to CSF^6^. The mutual fluid/solute exchange between ISF and CSF, both separated from the bloodstream by the blood–brain barrier (BBB) and the blood-CSF (B-CSF) barrier, is important for the elimination of waste and metabolic byproducts from the brain, being mediated by several mechanisms including diffusion, ion-channel transport, and hydrostatic/osmotic pressure-driven transport^7–9^. As to the mechanisms that move interstitial solutes through the dense neuropil, astrocytic aquaporin-4 (AQP4) channel-mediated water transport (known as ‘glymphatic’ transport)^7,10^ and the diffusion of solutes within the ISF^11,12^ have been identified as the major contributing factors. Physiological factors such as sleep and physical activity were also found to modulate the degree of transport; for example, slow-wave-stage sleep has been shown to increase the interstitial space^13^ while voluntary wheel running elevates glymphatic transport in mice^14^.

Other than the contributions from AQP4-mediated water transport and passive diffusion of solutes, convective bulk flow of CSF along the perivascular space (PVS) is another important transport element in driving solutes away from the brain. The PVS lines the cerebral vasculature in an intricate network and serves as an important passage for CSF transport, whereby pressure gradients generated by arterial pulsation (also referred to as ‘perivascular pumping’^15,16^) generate convective CSF flow and an accompanying advective solute movement that also facilitates interstitial/CSF solute movement in an AQP4-independent fashion^11,17,18^. Recently, a two-photon in vivo imaging study revealed striking visual evidence of advective movement of exogenous particles through the PVS adjacent to the pial arteries in mice^19^. Although the exact routes are still under intense investigation, it is thus far understood that the solutes/waste exit the brain via several pathways such as arachnoid granulation (exiting to meningeal venous sinuses), nasal mucosa, or meningeal lymphatic vessels (exiting to the cervical lymph nodes)^6,9^.

Controlled modulation of homeostatic transport of brain solutes by regulating AQP4 activities or promoting passive diffusion of the CSF solutes would be extremely challenging with currently available technology. However, generation of pressure-driven convective bulk flow in the brain is attainable, which led us to seek after a non-invasive technique that enhances the transport and clearance of interstitial solutes from the brain. Application of ultrasound waves imposes non-thermal radiation forces on the target media that induce directional flow. This phenomenon, known as the acoustic streaming effect, has been applied to deliver infusates to the brain by integrating an ultrasound-generating source to the injection needle/cannula in the context of convection-enhanced delivery^20,21^. Raghavan theoretically proposed the use of acoustic streaming to promote advective motion of various brain solutes in a non-invasive fashion^22^.

Focused ultrasound (FUS) techniques can deliver converged acoustic pressure waves to biological tissues-of-interest, using ultrasound transducer geometry, an acoustic lens, or through actuation of a multi-element transducer^23^. The use of a low ultrasound frequency range (on the order of 200–700 kHz, being lower than those used in ultrasound imaging) allows for ultrasound transmission through intact skull in a completely non-invasive fashion^24^. With its exquisite spatial selectivity and depth penetration in the brain, transcranial FUS (tFUS) has been used in a wide range of applications, such as ablation of brain tissue using a high-intensity ultrasound^25^, modulation of regional brain function via nonthermal low-intensity^26^, and delivery of high molecular weight (M_W_) drugs to the brain by disrupting the BBB when combined with microbubble contrast agents^27^.

Recent investigations have revealed that pulsed application of low-intensity FUS through the rat skull can enhance the transport of CSF tracers (intracisternally injected ovalbumin, OA) and deliver high M_W_ drug molecules (panitumumab; 150 kDa MW) to region-specific brain tissue, without disrupting the BBB^28,29^. These studies offered a promising potential of tFUS for enhancing CSF solute transport; however, open questions remain regarding whether it can enhance the transport as well as clearance of intracortically injected solutes from the brain. To address these questions, we injected fluorescent solute tracers having different M_W_ (45 kDa OA and 2000 kDa fluorescein isothiocyanate (FITC)-dextran, FITC-d) to the rat brain cortex and applied non-thermal low-intensity FUS to the site of injection. The extent of tracer distribution affected by the sonication was subsequently examined. Furthermore, to examine if the FUS can enhance the clearance of interstitial solutes out of the brain, we conducted a separate experiment to quantify the degree of OA drainage to the cervical lymph nodes (cLNs) promoted by FUS.

## Results

### FUS-mediated enhancement of tracer transport

**Figure 1:**
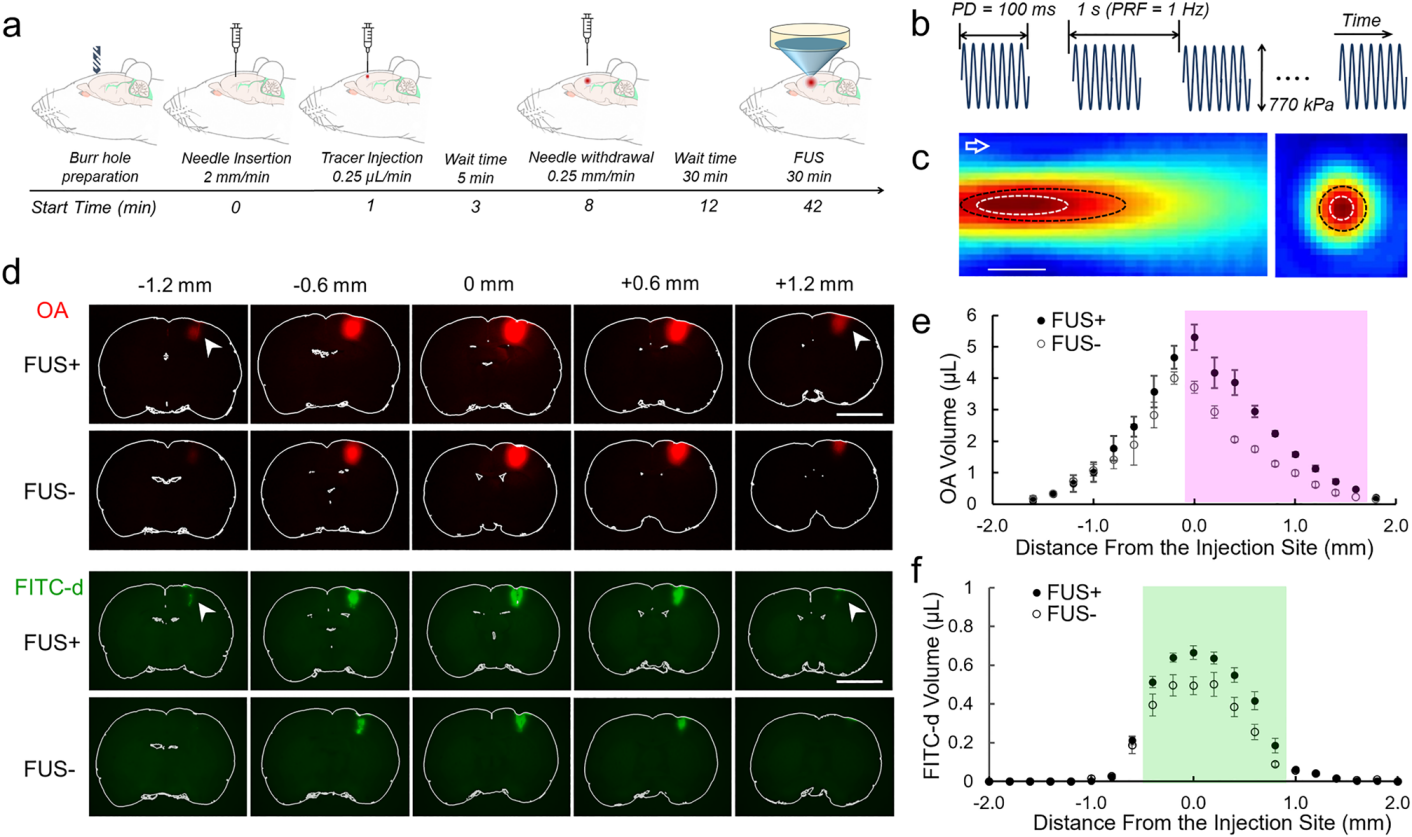
Transport of intracortical fluorescence tracers. (**a**) The schematic illustration of animal experimental procedures (start time = 0 min with respect to the needle insertion). Blue cone illustrates the acoustic beam profile from the FUS transducer. (**b**) Illustration of the ultrasound pulsing scheme. (**c**) The acoustic pressure profile along the longitudinal axis to the sonication (left) and the transverse plane at the focus (right). The arrow indicates the sonication direction. An acoustic focal profile bound at full-width-at-90%-maximum pressure (FW_90%_M) is shown by the white dotted line and the profile of full-width-at-half-maximum (FWHM) pressure is shown by the black dotted line (bar = 10 mm). (**d**) Exemplary OA and FITC-d distributions from a rat. 0 mm in the *x*-axis denotes the injection site while positive direction of the axis indicates rostral direction. (**e**) Slice-specific volume distributions of OA and (**f**) FITC-d tracer. The shaded areas represent the slices showing a significant difference (*P* < 0.05) between the FUS + and FUS− conditions (n = 7 each). Error bar: standard error of the mean (SEM).

The experimental procedure is illustrated in Fig. 1a. After exposing the dura through burr hole drilled through the skull, 0.5 µL of 45 kDa Texas Red OA and 2000 kDa FITC-d, separately constituted at 0.5 wt% concentration in artificial CSF (aCSF), were intracortically injected (3 mm right and 1 mm caudal to the bregma, 2 mm depth) to male Sprague–Dawley rats (n = 28) under ketamine and xylazine anesthesia. To prevent the effects from aggregation of OA in a FITC-d solution, the tracers were injected to separate groups of animals (n = 14 each).

FUS, operating at 200 kHz, was then stereotactically delivered to the injection site 30 min after needle withdrawal in a pulsed manner (100 ms pulse duration (PD) and 1 Hz pulse repetition frequency (PRF)) for 30 min at a spatial-peak pulse-average intensity (I_SPPA_) of 5 W/cm^2^ (10% duty cycle, 500 mW/cm^2^ spatial-peak temporal-average intensity—I_SPTA_) (the pulsing scheme was illustrated in Fig. 1b). The set of pulsing parameters, including acoustic intensity, was chosen based on the parameters that have been shown to enhance the transport of CSF solute in rats without inducing tissue damage or disrupting the BBB^28^. The use of 200 kHz frequency is applicable for transcranial delivery of ultrasound in humans due to lower skull insertion loss of acoustic energy compared to the use of higher frequencies^30^. The pressure at the focus, measured in degassed water, was 770 kPa (peak-to-peak amplitude) with a corresponding Mechanical Index (MI) of 0.86, which was much lower than the regulatory limit value of 1.9 that is currently defined for diagnostic ultrasound imaging of organs in the absence of gas-bodies in adults^31^. An acoustic focus, 5 mm in diameter and 15 mm in length, was defined by the profile bound at full-width-at-90%-maximum pressure. At full-width-at-half-maximum (FWHM), the acoustic focal size was 7 mm in diameter and 27 mm in length (Fig. 1c). The maximum pressure within the focus formed 11 mm away from the exit plane of the transducer, and the center of the focus was placed at the coordinates of the injection using a 3-axis robotic stage.

The weights of the animals were indifferent across the groups (Kruskal–Wallis H test; χ^2^(3) = 1.06, *P* = 0.79). As heart rate and anesthetic depth are factors known to affect CSF transport^19,32^, heart/respiratory rates, as well as peripheral oxygen saturation (SpO_2_) levels, were measured every 15 min at the onset of the experimental conditions. These variables did not change over time and were not different between the conditions (all *P* > 0.06, Supplementary Table S1).

Fluorescence images of 200 µm-thick brain sections that encompass the injection site showed that intracortically injected OA and FITC-d were transported to a greater distance from the injection site by FUS (noted as ‘FUS + ’) compared to the unsonicated control group (‘FUS−’) (the areas shown by the arrowheads, in Fig. 1d). Based on the analysis of distribution volume of OA, FUS conditions yielded 40.1% higher OA dispersion compared to the unsonicated control condition (FUS + : FUS− = 32.5 ± 6.8: 23.2 ± 4.1 µL, mean ± standard deviation, one-tailed, Mann–Whitney test, Z-score (Z_s_) = 2.36, *U* = 43.5, *P* = 0.009). Similarly, a 34.6% greater FITC-d distribution was observed in the FUS condition (FUS + : FUS− = 3.5 ± 0.4: 2.6 ± 0.6 µL, one-tailed, Mann–Whitney test, Z_s_ = 2.56, *U* = 45, *P* = 0.005). Within each condition (FUS + or FUS−), OA distribution volume was greater than FITC-d, which indicates the presence of size-dependent transport that favors the transport of small M_W_ OA (one-tailed, Mann–Whitney test, Z_s_ = 3.4, *U* = 49, *P* = 0.0002 within the FUS + , and Z_s_ = 3.07, *U* = 49, *P* = 0.001 within the FUS-).

The fluorescence images from contiguous brain sections across the animals were aligned with respect to the injection site, and tracer volume was estimated using automated criteria (described in ‘Fluorescent image acquisition and analysis’ in the “Materials and methods” Section). Slice-by-slice comparisons between the volume of OA and FITC-d uptake (in µL, shown in Fig. 1e,f, respectively) also reflected an increased volume of both tracers by application of FUS, whereby the difference was more profound toward the rostral side from the injection site (shaded areas indicate the slices showing a greater tracer volume in FUS + at *P* < 0.05, one tail Mann–Whitney test). We did not, however, find any notable ventral directional bias from the injection site (along the direction of acoustic propagation), where transport of the tracer occurred radially.to the injection site. In the control condition (FUS-), both tracers exhibited more symmetric distributions around the injection site.

### FUS-mediated enhancement of OA tracer clearance to the cervical lymph nodes

**Figure 2:**
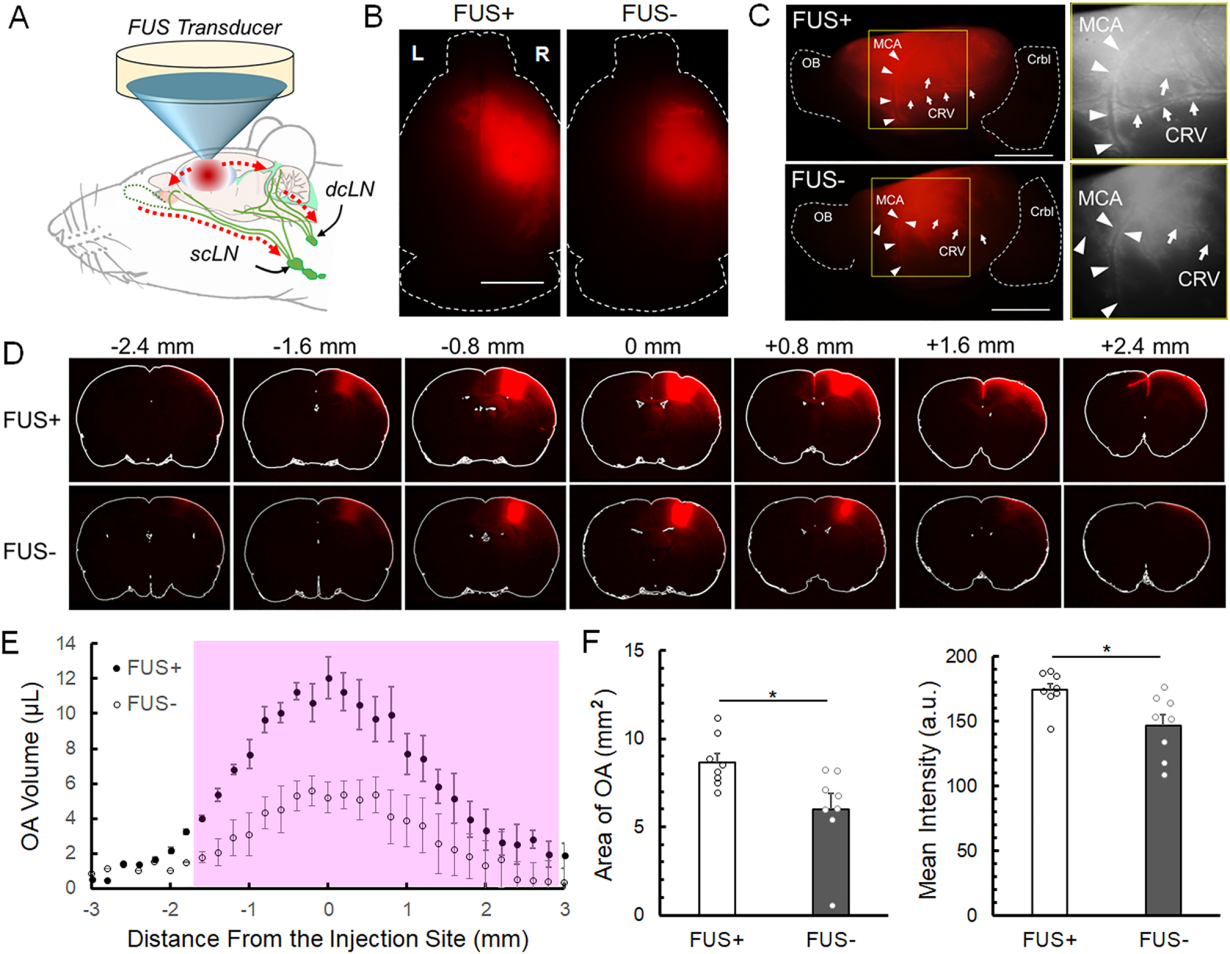
Transport of intracortically injected ovalbumin tracer. (**A**) Illustration of solute drainage to the deep cLN (dcLN) and superficial (scLN) by the application of FUS. Dotted lines denote the potential routes of drainage. (**B**) Examples of fluorescence images from the dorsal surface between FUS + and FUS− conditions and (**C**) corresponding fluorescence image ipsilateral to injection site, OB: olfactory bulb, Crbl: cerebellum, MCA: middle cerebral pial surface artery (indicated by arrowheads), CRV: caudal rhinal pial surface vein (indicated by arrows), bar = 5 mm. The yellow square areas were magnified in the grayscale image on the right. (**D**) Exemplary OA distributions of the brain slices between the FUS + and FUS- conditions, bar = 5 mm. (**E**) Slice-specific volume distributions of OA. 0 mm in the *x*-axis denotes the injection site while + indicates rostral direction. The pink shaded areas represent the slices showing a significant difference (*P* < 0.05) between the conditions (n = 8 each). (**F**) Comparisons on the area of OA distribution (in mm^2^) and the fluorescence (in arbitrary unit, a.u.) between FUS + and FUS− conditions. The circles indicate the individual values from 8 animals.

Because the interstitial transport of high M_W_ FITC-d was limited even with application of FUS (based on the results from the first study segment), only OA was used in the subsequent study assessing the interstitial tracer clearance to the cLNs. To increase the fluorescence detection sensitivity in the cLNs, 2 µL of higher concentration OA (1 wt%) in aCSF was injected into the same location in SD rats (n = 16). The hemispheric side of the injection was randomized and balanced. Upon 30 min wait time after needle retraction, FUS was delivered to the injection site for 1 h to allow for clearance of OA from the brain to the cLNs (FUS + , n = 8, illustrated in Fig. 2A, with routes of solute drainage to the cLNs). The rest of the animals (n = 8) did not receive sonication, constituting the control condition (FUS−). The weights of the animals were indifferent between the groups (two-tailed, Mann–Whitney test, Z_s_ = 0.47, *U* = 27, *P* = 0.64). Heart/respiratory rates, as well as peripheral oxygen saturation (SpO_2_) levels, remained unchanged over time and did not vary between the FUS conditions (all *P* > 0.25, Supplementary Table S2).

We found greater OA volume distribution from the dorsal brain surface imaging (an example shown in Fig. 2B, all images shown in Supplementary Fig. S1). The lateral surface fluorescence imaging from the tracer injection also reflected a wider OA distribution impacted by the sonication, with OA abutting the side of the pial surface vasculatures, which include the middle cerebral artery (MCA) and the caudal rhinal veins (CRV) (Fig. 2C, indicated with arrowheads and arrows, respectively, n = 5 shown in Supplementary Fig. S2).

From the examination of brain sectional images, a greater volume (i.e., 2 µL) and a longer sonication/monitoring time (60 min) yielded wider OA uptake to the brain parenchyma, encompassing ± 3 mm rostral/caudal side of the injection (Fig. 2D). Application of FUS resulted in a greater degree of volume distribution of OA compared to the unsonicated controls, with distribution volume of OA being ~ 125% greater than the control (FUS + : FUS− = 173.9 ± 59.8: 77.2 ± 64.57 µL, Mann Whitney test, one-tail, Z_s_ = 1.93, *U* = 40, *P* = 0.026). Slice-by-slice comparisons of the tracer volume (in µL) showed that sonication enhanced the interstitial transport of the OA with spatial bias toward the rostral direction of the injection site (Fig. 2E, shaded area, at *P* < 0.05, one tailed Mann–Whitney test). This greater volume of tracer uptake was supported by dorsal brain surface images showing ~ 43% greater area (FUS + : FUS− = 8.6 ± 1.4: 6.0 ± 2.4 mm^2^, one-tailed, Mann–Whitney test, Z_s_ = 2.36, *U* = 55, *P* = 0.009) as well as ~ 19% higher fluorescence intensity (FUS + : FUS− = 174.2 ± 14.3: 146.6 ± 24.4 *a.u.*, one-tailed, Mann–Whitney test, Z_s_ = 2.43, *U* = 55, *P* = 0.007, Fig. 2F).

**Figure 3:**
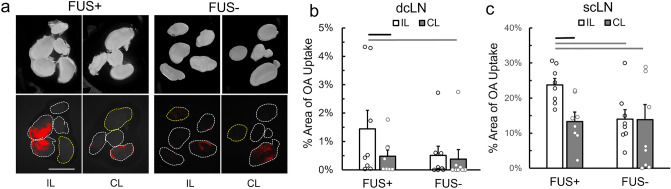
Drainage of intracortically injected ovalbumin tracer to cervical lymph nodes. (**a**) (top row) Bright-field images of extracted cLNs from FUS + and FUS− conditions and (bottom row) corresponding fluorescent images overlaid on thresholded OA fluorescence (in red). The contour of dcLN is marked in yellow dotted line while scLN is marked in white dotted lines. IL: Ipsilateral to FUS/injection site, CL: Contralateral to FUS/injection site. Bar = 5 mm. (**b**) The percentage area of the OA fluorescence from the dcLNs and (**c**) from the scLNs, measured from the FUS + and FUS− conditions (each from n = 8 animals). Black line indicates *P* < 0.05 from the Wilcoxon Signed-Rank test and gray lines indicate *P* < 0.05 from the Mann–Whitney test.

The number (ranging between 3 and 6) and areas (33.1–77.5 mm^2^) of lymph nodes were indifferent between FUS + and FUS- conditions as well as between the sides of injection (two-tailed Mann–Whitney test, all *P* > 0.05, detailed information in Supplementary Table S3 and Fig. S3). Application of FUS resulted in higher OA uptake in the cLNs (exemplar image shown in Fig. 3a) while the % uptake in the deep cLN (dcLN) was lower than that in the superficial (scLN) (one-tailed, Mann–Whitney test, Z_s_ = 6.23, *U* = 54, *P* < 0.001). In the FUS + condition, the area of OA uptake in the dcLN was significantly greater from the side ipsilateral to sonication (IL, 1.4 ± 1.8%) than that of the contralateral side (CL, 0.5 ± 0.6%; Fig. 3b, one-tailed, Wilcoxon Signed-Rank test, Z_s_ = 1.76, *P* = 0.039, n = 8) as well as that of the side contralateral to injection during control condition (FUS−, 0.4 ± 0.9%, one-tailed, Mann–Whitney test, Z_s_ = 1.96, *U* = 53, *P* = 0.02). However, this difference was not observed with respect to the OA uptake in dcLN ipsilateral to injection from the control animals (0.5 ± 0.9%, two-tailed, Mann–Whitney test, Z_s_ = 1.21, *U* = 44, *P* = 0.23).

In scLN, FUS yielded 78.3% higher uptake of OA to the injected side (23.7 ± 5.2% area, Fig. 3c) compared to the contralateral side (13.3 ± 6.6%; one-tailed, Wilcoxon Signed-Rank test, Z_s_ = 2.42, *P* = 0.008, n = 8). The degree of OA uptake was also higher than both sides of scLN obtained from the control condition (FUS−, IL: CL = 14.0 ± 7.6: 10.8 ± 12.2%, one-tailed, Mann–Whitney test, Z_s_ = 2.56, *U* = 56, *P* = 0.005 and Z_s_ = 1.96, *U* = 51, *P* = 0.02, respectively). Within the control condition (FUS-), there was no difference in the percentage area of OA uptake between the sides of OA injection (two-tailed, Wilcoxon Signed-Rank test, Z_s_ = 0.2, *P* = 0.2, n = 8). We note that tracer uptake in both dcLN and scLN showed large variations in OA drainage within the group, especially in dcLN where apparent OA drainage was not detected in some animals.

### Electroencephalogram (EEG) measurement and electrophoresis of fluorescent tracers

**Figure 4:**
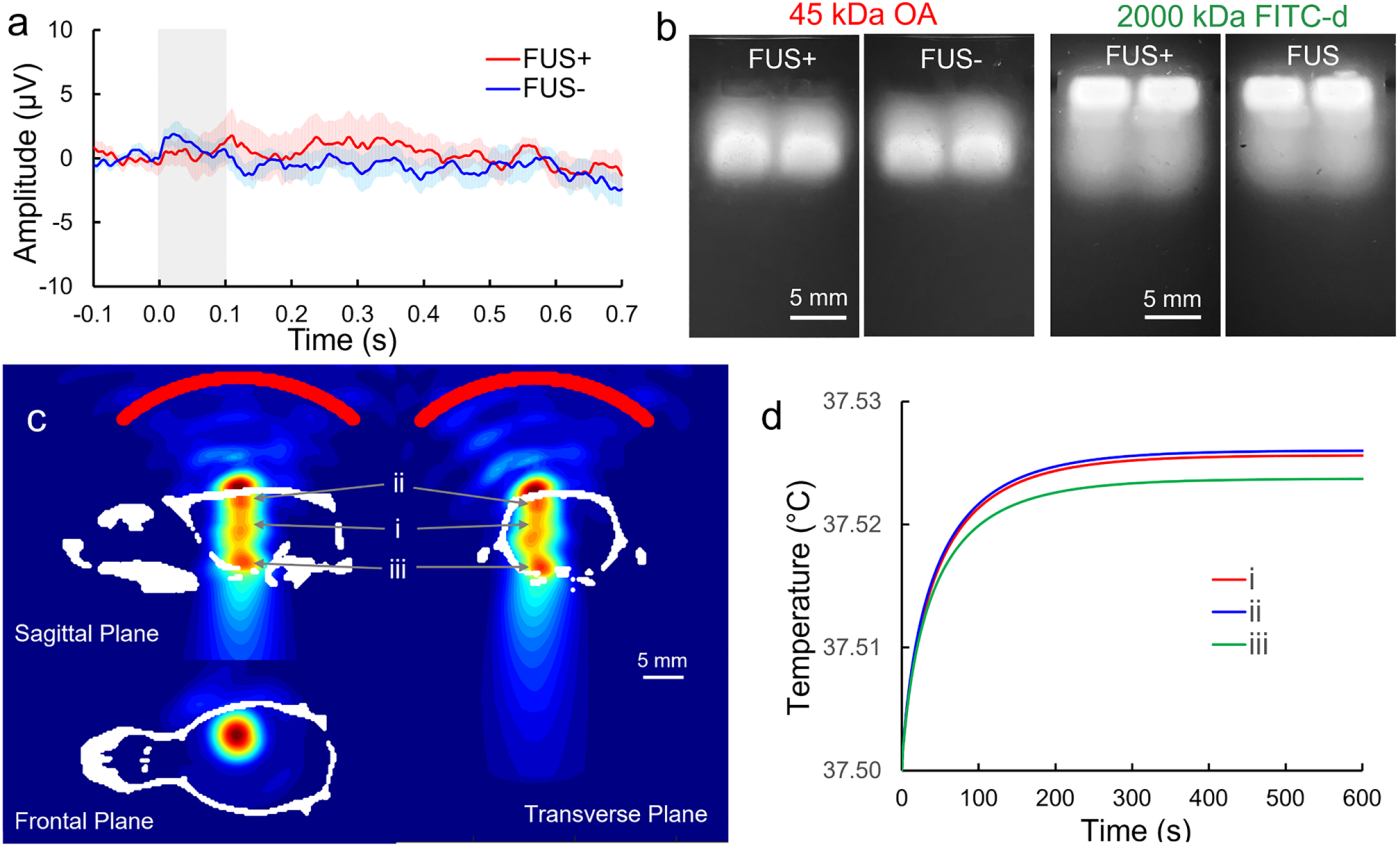
Evaluation of EEG features from the rats, molecular weight of the tracers, and evaluation of thermal effects of FUS. (**a**) Averaged EEG acquired from FUS condition (FUS + ; red line) and without sonication (FUS−; blue line) spanning 100 ms prior to the sonication onset to 700 ms after. The shaded area indicates the standard error (n = 9), and the gray box indicates the duration of sonication (100 ms). There was no statistical timewise voltage difference between the two conditions. (**b**) Gel electrophoresis of OA and FITC-d. Elution of each tracer was photographed in the same field-of-view using automatic exposure and cropped with white spaces. Uncropped full-length gel photographs are shown in Supplementary Fig. S4. (**c**) Pseudo-colored acoustic intensity map normalized with respect to the maximum value obtained from the numerical simulation was displayed in triplanes of acoustic focus (obtained from one rat skull, outlined in a white cranial profile). The profile of the transducer is marked by the red line. Bar = 5 mm. (**d**) Numerical estimation of tissue temperature at local maximum of (i) the geometric acoustic focus, (ii) the brain tissue interfacing the skull surface facing the incident sonication, and (iii) brain-skull base interface.

As the use of a specific acoustic parameter can also stimulate the brain^26,33,34^, EEG was measured from a separate group of animals (n = 9, weight = 285.7 ± 10.5 g) to evaluate whether the applied sonication resulted in neural activation. It was found that sonication did not elicit any EEG peaks distinguishable from the control condition (two-tailed *t*-test, *t*-score < 1.49; *P* > 0.16 across all time points) and the signal amplitudes remained within the noise level (± 3 µV; Fig. 4a), indicating the absence of sonication-induced brain stimulation.

We also performed electrophoresis on the two fluorescent tracers to examine whether sonication would alter their M_W_ distributions as dextran is a long-chain molecule. The smaller-M_W_ OA eluted farther than FITC-d, whereby most of the FITC-d fluorescence stayed within the well (Fig. 4b; full-length gel elution shown in Supplementary Fig. S4). A portion of the fluorescence from FITC-d was detected at a greater distance (9.5 ± 0.3 mm; n = 16) compared to the elution distance of the 45 kDa OA (8.9 ± 0.2 mm shown as a ‘trail’; one-tailed *t*-test, n = 16, *P* < 0.001), which suggested that FITC-d contained dextran molecules smaller than 2000 kDa. Nonetheless, there was no difference in eluting distance between the FUS− and FUS + conditions for both tracers (FITC-d, FUS-: FUS +  = 9.5 ± 0.3: 9.5 ± 0.3 mm; OA, FUS−: FUS +  = 8.9 ± 0.2: 8.8 ± 0.2 mm, n = 8 each group, two-tailed *t*-test, all *P* > 0.7), indicating that the sonication did not alter their M_W_.

### Numerical simulation of acoustic propagation and thermal analysis

The numerical simulation was performed using rat skull computed tomography (CT) data (n = 3) in two steps: (1) to estimate the in situ acoustic intensity distribution by modeling the propagation of acoustic waves in the rat cranial cavity and (2) to evaluate temperature of the brain tissue and skull-brain interfaces by solving the bio-heat transfer equation. The simulated spatial distributions of the acoustic intensity obtained from a rodent skull are shown at the triplane sections along the sonication path (Fig. 4c). In addition to the geometric focal area found at the injection site (I_SPPA_ 4.4 ± 0.2 W/cm^2^, peak-to-peak pressure of 679.6 ± 23.1 kPa, shown in Fig. 4c-i), we identified additional local maxima of intensity at the skull surface facing the incident FUS waves (I_SPPA_ 4.5 ± 0.4 W/cm^2^, peak-to-peak pressure of 698.1 ± 61.9 kPa, in Fig. 4c-ii) and at the brain tissue interfacing the skull base (I_SPPA_ 4.7 ± 0.4 W/cm^2^, peak-to-peak pressure of 722.2 ± 57.1 kPa, in Fig. 4c-iii, simulation results shown in Supplementary Fig. S5). The formation of additional local maxima, which yielded slightly higher intensities nearby the skull interfaces, was due to acoustic reverberations that are associated with the small size of rat cranium compared to the wavelength of the ultrasound (λ =  ~ 7 mm in water)^35^. Thermal analysis from these local maxima, despite the use of conservative overestimation of heat deposition, exhibited only a slight elevation of < 0.027 °C, reaching a steady-state maximum within 7 min (404 s) of sonication (Fig. 4d).

### Histological analysis

**Figure 5:**
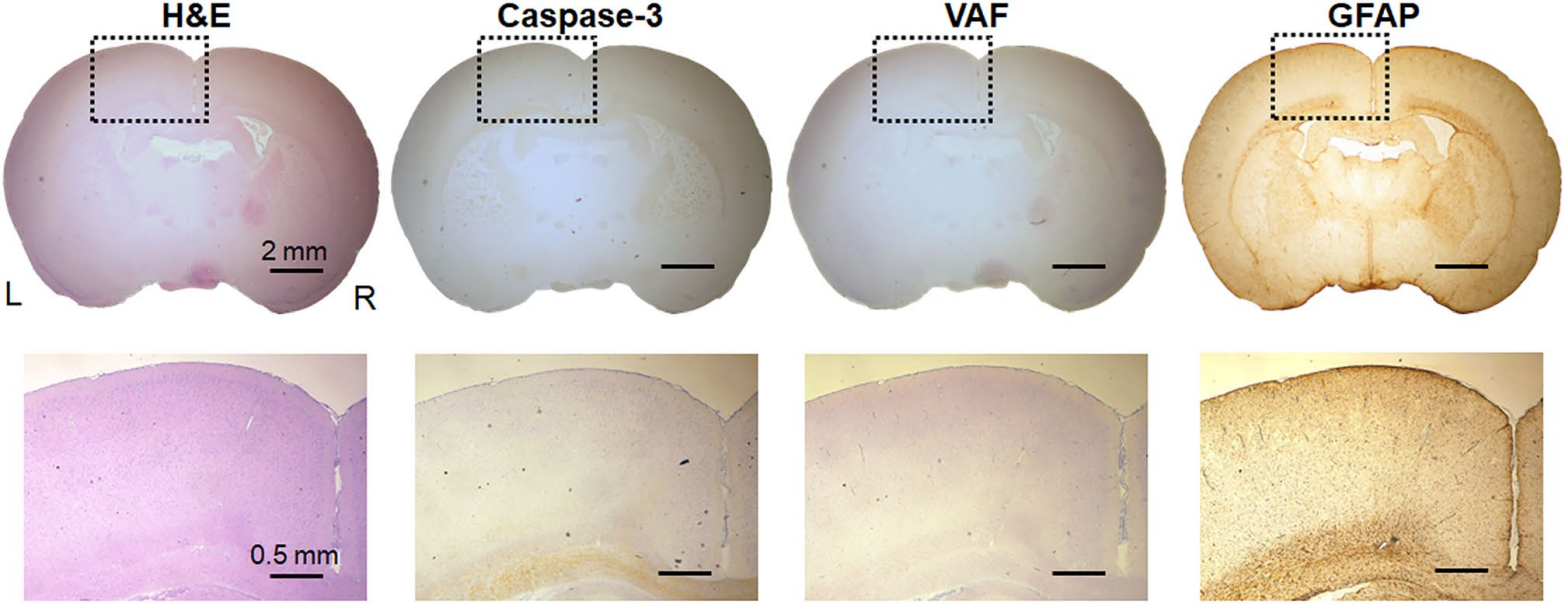
Exemplary histology of the brain tissues. Top row: H&E, caspase-3, VAF-toluidine blue, and GFAP (from left to right column) stained microscopic images of sonicated brain slices. bar = 2 mm, Bottom row: magnified images from the sonicated brain tissue (surrounded by the dotted rectangles). bar = 0.5 mm L = left; R = right.

The sonication parameters used in the present study have already been shown not to cause any damage to brain tissue through comprehensive histological analysis^28^; nonetheless, we assessed the effects of 1-h long sonication applied to the same coordinate of tracer injection (but without performing injection) in two male SD rats (weighing 270 and 275 g). The animals’ behavior was monitored during the 1-week survival period (20 min observation performed every other day). Both animals showed normal behavior, and the histological analysis of the sonicated brain regions did not reveal any signs of tissue damage (exemplary data shown in Fig. 5) in terms of global tissue integrity/hemorrhaging (from hematoxylin and eosin [H&E]), apoptotic activity (via caspase-3 immunohistochemistry [IHC] staining), ischemic damage (via vanadium acid fuchsin [VAF]-toluidine blue), and glial infiltrations (via glial fibrillary acidic protein [GFAP] staining).

## Discussion

Mounting evidence has emerged supporting the link between aberrant brain solute clearance and various neurological conditions such as sequelae of traumatic brain injury^36,37^, idiopathic normal pressure hydrocephalus^38^, stroke^39^, and neurodegeneration^40^. Non-invasive means to enhance solute transport from the brain parenchyma may provide novel therapeutic opportunities by enhancing the clearance of unwanted waste from the brain. Ultrasound sonication in combination with intravenous administration of microbubble (MB) contrast agents temporarily opens the BBB, allowing for the delivery of macromolecules across the BBB for various therapeutic purposes^27,41^. The technique has also shown to promote interstitial water transport mediated by upregulating AQP4 expression^42^ or amyloid beta (Aβ) clearance from the brain parenchyma to CSF in animal models^43^. Despite its promising potential in enhancing solute clearance from the brain, conjunctional use of MB-FUS accompanies a risk of local hemorrhaging by excessive BBB disruption induced by inertial cavitation^44,45^. In the present study, we demonstrated that transcranial application of FUS, given at a pressure level that does not cause BBB disruption (peak-to-peak amplitude of 770 kPa)^28^ without using any MB, not only promoted the transport of intracortically injected fluorescent tracers, but also facilitated their clearance from the brain.

### Enhanced transport of intracortically injected tracers

Comparing the distribution volumes between OA and FITC-d in the rat brain, we found that the smaller-M_W_ OA transported farther than the higher-M_W_ FITC-d, regardless of the presence of FUS. This finding agrees with the previous observation that the transport of interstitial solutes is size-dependent^11^. Interestingly, this dependency may suggest the presence of a diffusional component in interstitial solute transport being mixed with the convective transport^46,47^. A debate over the dominant mode of solute transport within the ‘true’ interstitial space (distinguished from neurovascular structures, including the PVS) between advection (accompanying the convective flow) and diffusion, has not yet been resolved, and our observation may indicate the contribution of two modes of solute transport that work in unison.

Slice-by-slice examination of tracer distribution in the brain revealed that FUS enhanced the transport of both OA and FITC-d, showing higher tracer distribution toward the rostral direction from the injection site. This indicates the presence of anisotropic interstitial/CSF solute transport. However, the results differed from the caudal bias reported in solute transport, which has been shown by the movement of gadolinium immunoglobulin accompanying intrathecal hyperosmolar mannitol co-infusion^48^ or intraparenchymal Evans blue dye in rodents^49^. We postulate that the needle bevel which faced the rostral direction during injection might have introduced the spatial bias in transport, however, the contribution of nasal brain lymphatic clearance pathways that are rostral to the brain is also likely^50,51^. It is also noteworthy that the enhancement of tracer transport occurred radially from the injection site rather than along the direction of wave propagation (i.e., toward ventral direction), which shared similarities to the one observed during ultrasound-assisted convection enhanced delivery (UCED) of Evans Blue dye in the rodent brain^52^. We surmise that anisotropic arrangement of perivascular space, for example, the interface between the cortex and the corpus callosum ventral to the injection site, might have reduced the directionality of solute transport. The presence of intracranial reverberation, as shown from our numerical simulation (Fig. 4c) could also contribute to reduced directionality of the transport along the sonication path. Further investigation is needed to uncover the exact routes and direction of brain lymphatic clearance across the brain volume.

We note that sonication greatly enhanced the transport of the intracortically injected FITC-d in the present study. This finding contrasted to our previous observation whereby the transport of intracisternally injected CSF FITC-d was not affected by FUS^28^. We hypothesize that the difference was attributed to the local concentration difference of the tracers exposed to the sonication path in which most of the intracortically injected (thus undiluted) FITC-d tracers were directly exposed to acoustic pressure field, thus propelled by the FUS to a greater distance than the intracisternally injected tracers that would be significantly diluted with the CSF.

### Enhanced clearance of intracortically injected OA tracer to cLNs

To allow for detectable clearance and subsequent drainage of the interstitial tracers to the cLNs, we injected a greater amount of OA (20 µg) in the second set of the experiment and doubled the sonication time (to 60 min). As anticipated, OA was transported farther compared to the first experiment (Fig. 2B,D), with the same rostral directional bias seen during the first segment of the study (Fig. 2E). From the examination of OA drained to the cLNs in the absence of sonication (FUS−), the side of OA injection did not have any impact on clearance to the cLNs, as shown by the equivalent OA uptake from both cLNs ipsilateral and contralateral to the injection. However, application of FUS resulted in distinctively higher OA uptake in the cLNs, especially in the scLN ipsilateral to sonication (Fig. 3).

FUS-mediated enhancement of OA drainage was also evident from the ipsilateral dcLN while the percentage area of tracer was much lower than that from the scLNs. This preferential drainage of OA to the scLN shared similarity to a previous work probing the movement of Evans blue dye observed after intra-striatum injection in mice^49^. This finding, however, was different from other studies that have shown the preferential dcLN clearance route of interstitial tracers and exogenous immune cells (T cells) through a network of dural lymphatic vessels^53,54^. As to the cause for this discrepancy, we surmise that OA, distributed with the spatial bias toward rostral part of the brain, was transported dominantly through the nasal lymphatic system having greater connectivity to the scLN than to the dcLN^50^. A portion of the interstitial OA transported to the pial surface vasculature (visible in Fig. 2C) and is collected at adjacent dural lymphatic vessels, such as middle meningeal arteries^53,55^, and then drained to the dcLN, albeit in a lesser extent than scLNs.

In addition, we found large within-group variations in OA drainage to the cLN (Fig. 3), especially in the dcLN, where fluorescence was not detected in some animals. The time between the tracer injection and the organ harvest was maintained across the animals and group; thus, the tracer collection time would not have played a major role in the observed variations. Although we cannot ascertain the causes for this finding, we surmise that individual variations in the efficiency of brain solute transport (e.g. through glymphatic and/or periarterial transport) may have ramified into different solute transit times to the cLNs. A longer wait time (than 1 h used in in present study) may eventually allow for collection of tracers across all animals. As we cannot completely rule out the contribution of other routes of solute clearance (i.e., drainage to meningeal venous sinus via arachnoid granulations or choroidal plexus), further studies are needed to understand the detailed lymphatic exit routes, for example, through large animal models (e.g., pig or sheep) which have more visible meningeal arachnoid granulations. Studies in large animals are also attractive since their larger skull dimensions would significantly reduce the contribution from acoustic reverberation that was seen in the present study.

### Potential mechanisms

To our knowledge, the present findings represent the first evidence that pulsed application of FUS can regionally enhance not only the transport, but also the clearance of brain interstitial solutes. The M_W_ of the OA used in this work was comparable to those of neuro-reactive Aβ oligomers (~ 8–70 kDa)^56^, and the enhanced level of OA drainage suggests promising future therapeutic potential of tFUS in the removal of Aβ. Heart rate, which may affect PVS pulsation (and associated solute transport) as well as other vital signs such as respiratory rate and SpO_2_ which reflect the depth of anesthesia, were found to be indifferent across the conditions, indicating that our results were not confounded by these physiological variables. Through numerical simulation of acoustic propagation and thermal analysis (Fig. 4c,d), we found that application of FUS, due to the low intensity used, would not heat the brain tissue. Although we postulate that the acoustic streaming conferred by pulsed FUS served as the main driving force behind the enhanced transport and clearance of interstitial solutes, the underlying mechanism remains unclear, as the presence of acoustic dispersion and complex acoustic interactions with poroelastic brain tissue may also contribute to advective solute movement^52^. which are subsequently collected by meningeal lymphatic vessels and nasal lymphatics.

We also note that a degree of microscopic disruption between the PVS and neuropil present during the direct tracer injection may render a portion of the tracers to leak into the PVS (and consequently be transported by FUS). Although the intracortical injection technique is widely adopted in studying the movement of interstitial solutes, glial cell damage that may accompany the needle injection may also acutely reduce AQP4 channel function^49^, reducing tracer transport. Experimentation on animals that do not require injection of exogenous tracers, for example, on a rodent AD model and subsequent quantification of FUS-mediated drainage of endogenous Aβ proteins (achievable by the positron emission tomography), may address the contribution from these confounders.

The FUS parameters used in this work, i.e., 100 ms PD given in 1 Hz PRF, did not alter EEG brain activity (Fig. 4a). This is consistent with evidence of inefficient brain stimulation associated with the use of a long acoustic pulse duration^57^. However, cell-level neuronal activity induced by sonication may present, which may subsequently impact brain solute clearance. In addition, it is possible that the sonication itself might have altered the level of AQP4 expression affecting the interstitial water transport^58^. Thus, assessment of cell-level function/expression of the AQP4 or neuronal activity (e.g., c-Fos) affected by FUS would offer further insight on the underlying mechanism behind our observation.

We acknowledge that optimization of the sonication parameters is also warranted to maximize the effect of FUS at the lowest possible acoustic intensity (and pressure amplitudes). For example, a higher frequency than the one used in this study (i.e., 200 kHz) can be adopted to enhance the transport at the same pressure level. In vitro dye infiltration experiments in a porous medium such as hydrophilic polyvinyl alcohol (PVA) foam (average pore size of 80 µm), possessing porous microstructures that approximately mimic the PVS (~ 40 µm diameter tubular PVS along each side of the arterial wall^19^), can be used to optimize sonication parameters prior to further in vivo applications. Numerical modeling of tracer behavior affected by FUS, in this regard, may additionally be sought after to estimate the interstitial solute movement. However, it is important to note that acoustic streaming behavior is highly nonlinear and depends on the sonication parameters and microstructure of the porous media^59^. For example, Eckart streaming is dominant in structures that are greater than the wavelength of the ultrasound, while Rayleigh streaming becomes dominant in structures smaller than the wavelength^60^. Since the detailed cytoarchitecture of the brain, including the PVS, is still unknown, numerical prediction of realistic fluidic movement (including solute movement) in the brain would be extremely challenging, yet constitute a subject for future investigation.

### Importance of post-tracer injection wait-time before FUS

Regarding the experimental technique, which involved a post-tracer injection wait time of 30 min prior to the application of FUS, we previously reported that the same intensity of FUS to the rat brain, applied a short time (e.g., 7 min) after intracortical injection of interstitial tracers, caused massive intracranial hemorrhaging in a significant portion of tested animals^61^. We did not, however, observe any hemorrhaging in the present experiment protocol, suggesting that the increased 30 min wait time prior to sonication allowed impaired cerebrovascular structure (caused by the needle injection) to close shut, preventing hemorrhaging.

### Technical limitations of the study

We note several technical limitations of our approach. The fixation method (i.e*.,* PFA perfusion), despite being considered the gold standard in tissue fixation, is known to reduce the volume of PVS^19^, and thus may decrease the observed fluorescence level in the brain tissue. Despite this limitation, the PFA perfusion used uniformly across the experimental groups would not alter the observed effects of FUS in enhancing the brain solute transport and clearance. Immersion fixation can be considered as an alternative; however, the long fixation time necessary for the relatively large rat brain (compared to the mouse brain)^62^ may generate even more undesirable artifacts while the impact of immersion fixation on the brain lymphatic system is not fully understood. Therefore, adoption of imaging techniques such as in vivo two-photon microscopy during sonication is highly desired to monitor interstitial tracer movement/distribution in live animals^19^. We also acknowledge that the current study was performed only in male rats, which necessitates experimentation on female rats to examine any gender-specific difference of the FUS effects as gender-dependent meningeal lymphatic vessel architecture was recently found in humans^63^.

## Conclusions

Efficient interstitial solute transport and lymphatic clearance of undesirable metabolic waste from the brain are vital to normal brain function. We demonstrated that transcranial application of FUS non-invasively promoted movement and drainage of intracortically injected solutes in rats. In humans, the brain area that requires waste clearance would not be limited to a single spot, for example, relatively large areas across neocortical regions show increased Aβ burden during the preclinical stage of AD^64^. Thus, in consideration of potential application to humans, a FUS system and transducer design should depart from a traditional mean of a single focal delivery of acoustic waves, and instead enable sonication of wide and irregular shapes of the brain volume-of-interest. Electronic steering of the FUS beam/focus is attainable using the phased-array transducer configuration^65^, but a simpler, mechanical steering of ultrasound can also be conceived as an alternative. Furthermore, 3D-printed acoustic lenses coupled to a piezo material can also be used to sonicate in the desired shape covering a much wider volume^66^. As brain waste clearance may require multiple sessions of FUS, the ergonomics of the transducer design for patient comfort will become an important factor. In summary, although understanding detailed mechanisms of waste elimination from the brain demands intensive future investigation, FUS, having the ability to regionally facilitate CSF/interstitial solute movement, possesses the much-needed non-invasive potential for controlled enhancement of waste transport, including elimination from the brain.

## Materials and methods

### Fluorescent tracers

45 kDa-M_W_ Texas Red OA (Thermo Fisher) and 2000 kDa-M_W_ FITC-d (Millipore Sigma) were used as interstitial fluorescent tracers. Since OA tends to aggregate in a FITC-d solution, the tracers were prepared separately, each being constituted at a 0.5 wt% concentration in artificial CSF (aCSF; Tocris Bioscience). In the second experiment assessing the degree of brain solutes clearance to the cLNs, only OA was used because the clearance of large-M_W_ FITC-d to the cLNs are minimal during ~ 2 h experimental period. To increase the detection sensitivity of OA drained to the cLNs, OA was prepared at an increased concentration (1 wt%) in aCSF.

### Animal procedures and intracortical injection

All animal procedures were approved by the Institutional Animal Care and Use Committee (IACUC) of the Brigham and Women’s Hospital and conducted in full compliance with its regulations and standards. All methods are reported in accordance with ARRIVE guidelines (https://arriveguidelines.org). Sprague–Dawley (SD) rats (all male, n = 46) were socially housed (two rats/cage) under a 12 h/12 h light/dark cycle (lights on at 7 AM, off at 7 PM) and were allowed access to food and water ad libitum. Rats were anesthetized using an intraperitoneal injection of a mixture of 80 mg/kg ketamine and 10 mg/kg xylazine. After reaching adequate anesthetic depth, the animal’s head was shaved using a clipper and depilation lotion. The animal was then placed on a stereotactic frame (ASI Instruments) while a 37 °C warming pad (Gaymar) was placed under the animal to maintain its body temperature. Anesthetic plane was assessed every 10 min by checking the reflex to toe pinching. Additional anesthetic doses (1/3–1/2 dose each) were administered as needed. Respiratory and heart rates, along with peripheral blood oxygenation (SpO_2_) measured using a pulse oximeter (Smiths Medical), were recorded starting right before the application of FUS (or the start of the control condition) and every 15 min until the end of sonication.

The prepared tracer solution was intracortically injected based on established surgical protocols^4,7^. After preemptive administration of 2% lidocaine hydrochloride under the skin near the incision, a midline incision was made to expose the skull to identify the bregma. A skull burr hole was drilled (Fine Science Tools) 3 mm lateral and 1 mm caudal to the bregma without puncturing the dura. Then, a 30-gauge (30G) needle (0.1 mL, Hamilton) was stereotactically inserted 2 mm deep at a rate of 2 mm/min. The direction of the needle bevel faced the rostral direction across all experimental conditions. Tracers were injected at a rate of 0.25 µL/min for 2 min (a total volume of 0.5 µL) using a syringe pump (KD Scientific). Following a 5 min waiting period, the needle was retracted at 0.5 mm/min for 4 min. In the experiment that examined the effect of FUS on the drainage of OA to the cLNs, 2.0 µL of tracer solution was injected at a rate of 0.25 µL/min for 8 min whereby the side of the injection (left or right hemisphere) was randomized and balanced across the animals.

### FUS transducer and acoustic field characterization

A FUS transducer (Ultran Group), operating at a 200 kHz fundamental frequency, was actuated by a sinusoidal electrical waveform from a function generator (Keysight) connected to a 40-Watt linear power amplifier (Electronics and Innovations). The spatial profile of the pressure amplitude was directly mapped with a 1 mm step resolution using a needle hydrophone (Onda Corp) mounted on a 3-axis robotic stage in a degassed water tank based on a method described in detail elsewhere^33^. The pressure at the acoustic focus was calibrated with respect the input voltage using a calibrated hydrophone (Onda Corp).

### Rodent FUS platform and application of FUS

The animal was positioned over an in-house built stereotactic robotic sonication platform (Newmark Systems) equipped with ear and bite bars. Planar movement of the FUS transducer, which was attached to a horizontal translation stage, was independently controlled with respect to the vertically moving platform on which the animal was placed. All motorized stages had a spatial precision of 15 µm. Prior to sonication, the site of intracortical injection was marked with a surgical pen to align the acoustic path to the injection site, and a ~ 8 mm gap between the scalp and the exit plane of the transducer was maintained to place the acoustic focus on the injection site (2 mm ventral from the scalp). The gap between the transducer and the scalp was filled with a compressible acoustic coupling gel that was prepared with 9% w/v aqueous polyvinyl alcohol solution (Millipore Sigma), which was molded through two 16 h–8 h freeze–thaw cycles. Acoustic hydrogel (Parker Lab) was applied to all interfaces.

FUS, delivered immediately following needle retraction, has previously been found to cause intracranial hemorrhaging^61^. Thus, a 30 min wait period was introduced after the needle retraction. The wait time has also been shown to prevent retrograde efflux of the tracer^67^. Then, FUS was stereotactically delivered to the injection site in a pulsed manner for 30 min (n = 7 in each group). The other seven animals (in each tracer group) did not receive any sonication, constituting a control condition (i.e*.,* ‘FUS-’ condition). In the experiment that examined the effect of FUS on clearance of intracortically injected OA, the same sonication parameters (n = 8), was administered for 60 min to accommodate the time needed for the detectable drainage of intracortical tracer to the cLNs. Eight rats underwent the same procedure without receiving sonication (‘FUS−’ condition).

### Fluorescent image acquisition and analysis

Immediately after a FUS session (including the non-sonication condition in the control groups), necropsy was performed to harvest the animal’s brain via transcardial perfusion of 4% paraformaldehyde (PFA) in phosphate buffered saline (Boston Bioproducts). The time between the tracer-injection to the completion of transcardial perfusion (~ 30 min) was maintained across all animals. The extracted tissue underwent an additional 24 h submerge fixation in PFA. In the second experiment that examined the effects of FUS on OA drainage, both dcLN and scLN were removed. The brain was cut in a 6 mm-wide block encompassing the acoustic focus and further sectioned in 200 µm-thick slices using a vibratome (Ted Pella) along the rostral-caudal direction. The sectioned slices, dorsal brain surface, and lymph nodes were imaged with a widefield fluorescent microscope (Nikon) using an ultrawide-field (23.4 mm × 15.6 mm) CMOS sensor (4592 px × 3056 px resolution, Sony). In the second set of experiments, the lateral surface of the brain was also imaged from a few rats (n = 5:5, FUS + : FUS−) to visualize the pattern of OA tracer distribution over the lateral pial surface vasculature. All images were separated by color channels into 8-bit images and resulting grayscale images with appropriate fluorescent emission were used for subsequent analysis.

To estimate the tracer volume (both OA and FITC-d) from each vibratome section, the location of sectioned images obtained from each animal were aligned with respect to the injection site (denoted as 0 mm) and thresholded above three times the mean absolute deviation of intensity distributions to delineate the pixels that show tracer uptake. The volume of tracer uptake in each section was then derived from the number of delineated pixels (image pixel = 4.33 pL). The area of OA distribution in the dorsal surface images of the brain were segmented using the mean value of automatic threshold levels (the isodata algorithm implemented in ImageJ^68^) applied to the images from the FUS + group. To calculate the percentage area of OA uptake to the cLNs, both extracted cLNs were placed under the same field-of-view of the microscope for imaging. To account for the lower fluorescence than the brain surface images, the area of OA uptake in the cLNs was segmented using 50% above the auto-threshold value from the image ipsilateral to OA injection. The area occupied by the cLNs was segmented from the bright-field image (21.6 µm^2^/pixel).

### EEG measurement during the sonication

To minimize the electrical artifact associated with proximal contact with the transducer (i.e., the transducer may have acted as a capacitor during pulsed sonication and generated a spike artifact), sonication was delivered to the brain ventrally through the neck using our previous technique^28^. Two subdermal wire EEG electrodes (Ives EEG Solutions) were inserted under the midline skin over the scalp with a ~ 10 mm gap, and a ground electrode was attached to an ear. EEG was measured every second, 300 times, in a time-locked manner (from − 100 to 700 ms windows with respect to the sonication onset) and subsequently averaged after applying a 100 Hz low-pass and Mains filters. EEG was also acquired from the same animal without the sonication, with the sequence of control and sonication conditions randomized across the animals.

### Electrophoresis of fluorescent dyes

150 µL of each tracer solution was placed at the acoustic focus (over a thin polyethylene terephthalate film for uninterrupted acoustic transmission; nominal thickness of 13 µm, absorption of acoustic energy was undetectable by hydrophone measurement) and sonicated using identical parameters as those given to the animals. The same volume of tracer was prepared without the sonication to provide a control condition. Immediately after sonication, 10 µL of both sonicated and unsonicated tracers (n = 8 each) were loaded to an agar gel (low-gelling temperature agarose, 0.8% by weight, Sigma) in a buffer solution (1:1 by volume = normal saline: distilled water) and were subjected to gel electrophoresis for 25 min (48 V constant voltage, Minipcr). The elution was then photographed.

### Numerical simulation acoustic propagation and thermal analysis

Information on three-dimensional geometry of skulls were obtained from ex vivo rat skulls (n = 3; without the mandibular bones) using computed tomography (CT, Source 60 kV, Bruker) for the numerical simulation of acoustic propagation within the cranium. CT images were acquired in isotropic voxels of 17 µm and were resampled to iso-voxel of 0.25 µm, yielding a ratio of 30 pixels per wavelength in water (λ =  ~ 7.5 mm at 200 kHz) to attain sufficient spatial discretization for the simulation^35^. The FUS source was modeled based on our previous method^69^ according to the transducer geometry (transducer diameter of 28 mm and the radius-of-curvature of 22 mm) and was positioned according to the sonication geometry used in the animal experiment. Linearized Westervelt-Lighthill equation for analyzing acoustic wave propagation was solved by the numerical scheme of finite-difference time domain (FDTD) in a volume encompassing both the transducer and skull (183 × 275 × 266 voxels), with a time resolution of 0.05 µs. Voxels in the image were expressed in Hounsfield unit (HU) values that are calibrated to a water tube located within the same image volume, and the acoustic properties of the skull (i.e., speed of sound, density, and attenuation) were used in the simulation, as described in our previous work^69^. Based on the resulting spatial distribution of acoustic intensity, the bio-heat transfer equation was subsequently solved using the FDTD method^70^. The time-series temperature change in the media was obtained in a time resolution of 10 ms, and the sonication parameters (I_SPPA_ of 5 W/cm^2^, 100-ms PD, 1-Hz PRF [i.e., 10% duty cycle] and a maximum of 1-h sonication duration) were used in the estimation assuming homogenous intracranial brain tissue at an initial temperature of 37.5 °C. Thermal properties of the brain (specific heat capacity *c* of 3600 J/kg/K, thermal conductivity κ of 0.528 W/K/m), skull (*c* of 1300 J/kg/K, κ of 0.4 W/K/m) and blood perfusion (*c* of 3620 J/kg/K, perfusion rate of 8.24 kg/m^3^/s, and density of 1030 kg/m^3^) were used as inputs in the simulation^71^. All simulations were performed in a graphic processing unit (GPU) environment with parallel processing. To conservatively overestimate the potential temperature rise in the rat brain tissue, contributions from thermoregulatory responses and the CSF pulsation that transfer/remove heat deposition^72^ were not modeled.

### Data analysis

A statistical analysis was performed using MATLAB software (Statistics and Machine Learning Toolbox, Mathworks). The normality and sphericity of data distribution was examined by Kolmogorov–Smirnov normality test and Mauchly's test, respectively. The Kruskal–Wallis H test was performed to examine differences in the animals’ weights among the groups. Repeated measures analysis of variable (ANOVA) was conducted to examine the presence of time-dependent changes in vital signs (heart/respiratory rates and SPO_2_) between the groups, separately for the type of tracers. Condition-specific differences in the volume/surface distribution, and percentage OA area in the lymph nodes were assessed using Mann Whitney test and Wilcoxon Signed-rank test (hemispheric comparisons within a group). The time-series EEG data and tracer eluting distance from the electrophoresis were assessed using a *t*-test. The statistical significance was set at *P* < 0.05.

### Histological analysis

Without performing surgery for the tracer injection, two rats were placed on the stereotactic frame, and sonication was delivered for 1 h to the same coordinate as the injection site in reference to the interaural line. The fur over the head was shaved prior to FUS. One week later, the rats were sacrificed via exsanguination and subsequent transcardial perfusion (4% formaldehyde). The sonicated brain area was coronally sectioned and stained with H&E (GHS-2–16, Sigma-Aldrich, St. Louis, MO) to detect necrosis or hemorrhaging, caspase-3 IHC (ab4051, Abcam, Cambridge, UK) to visualize apoptotic cells, VAF-toluidine blue (A3908, Sigma-Aldrich) to detect the presence of ischemic damage, and GFAP (ab7260, Abcam) to detect glia infiltration that may be associated with neuroinflammation. Chromogenic in situ hybridization for IHC was performed using a bond polymer refine detection kit (DS9800, Leica Biosystems, Buffalo Grove, IL) with citrate antigen retrieval based on a primary antibody dilution factor of 1:300 for GFAP and 1:500 for caspase-3.

## Supplementary Information

Supplementary Information.

## Data Availability

All data are included and presented through Supplementary Information. The datasets generated and analyzed during the current study are curated and the publicly available data repository of Harvard Dataverse. https://dataverse.harvard.edu/dataset.xhtml?persistentId=doi:10.7910/DVN/GM0OI6
