# Supplementary material for: Non-invasive enhancement of intracortical solute clearance using transcranial focused ultrasound
Source: Sci Rep. 2023 Jul 31;13:12339. doi: 10.1038/s41598-023-39640-2 (PMC10390479; doi:10.1038/s41598-023-39640-2)
Supplement: Supplementary file 1 — Supplementary Information. [file 41598_2023_39640_MOESM1_ESM.pdf]

Supplementary Information

Non-invasive enhancement of intracortical solute clearance using transcranial focused ultrasound

Seung-Schik Yoo[1,], Evgenii Kim[1], Kavin Kowsari[1], Jared Van Reet[1], Hyun-Chul Kim[1,2], Kyungho Yoon[3]

[1]Department of Radiology, Brigham and Women's Hospital, Harvard Medical School, Boston, Massachusetts, United States of America

[2]Department of Artificial Intelligence, Kyungpook National University, Daegu, Republic of Korea

[3]School of Computational Science & Engineering, Yonsei University, Seoul, Republic of Korea

Supplementary Figures

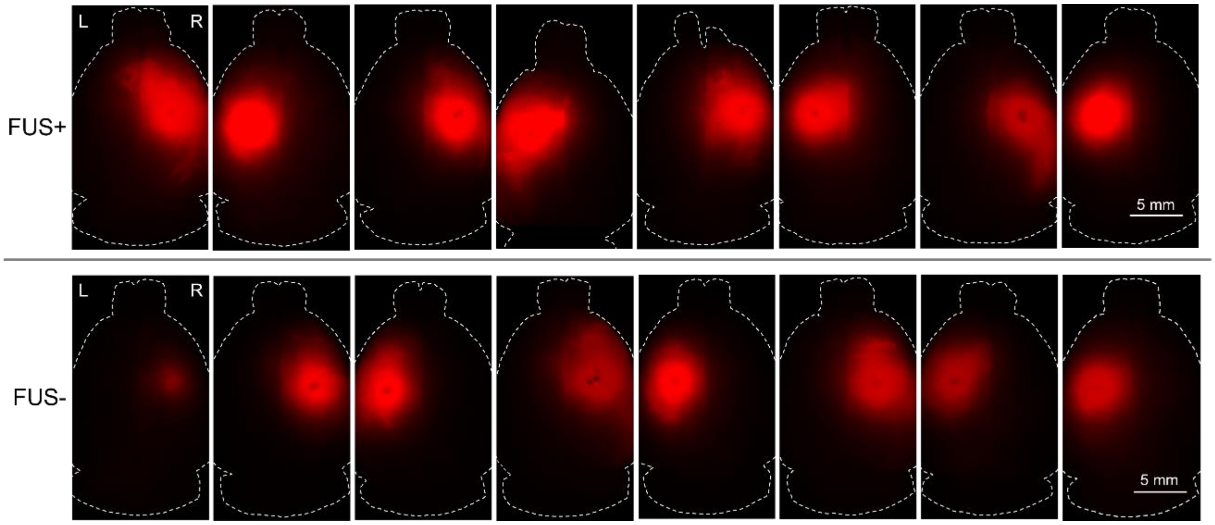

Figure S1. Dorsal surface fluorescence images of OA tracers (n = 8 in each group). The contour of the brain is delineated with dotted lines.

FUS+: FUS condition, FUS-: non-sonicated control condition, Bar = 5 mm

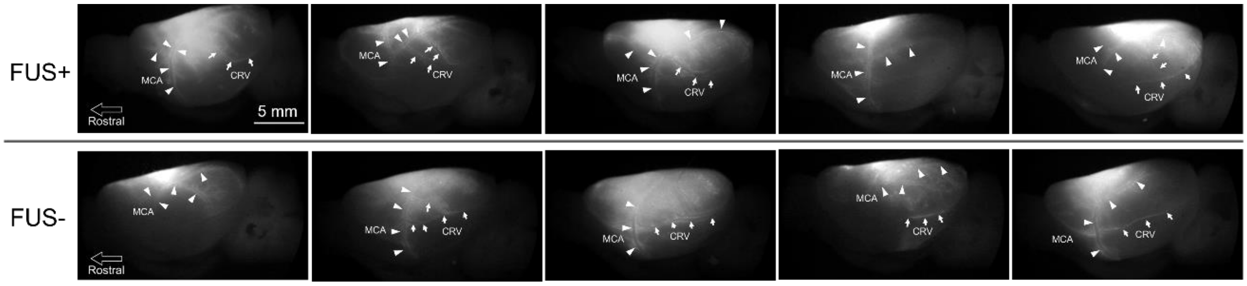

Figure S2. Grayscale surface fluorescence images, visualizing the pial surface vessels ipsilateral to OA injection (n = 5 in each group). No apparent fluorescence was seen from the hemisphere contralateral to the injection/sonication (not imaged). Arrows indicate the rostral direction of the brain.

MCA: middle cerebral artery (arrowhead), CRV: caudal rhinal vein (arrow). Bar = 5 mm

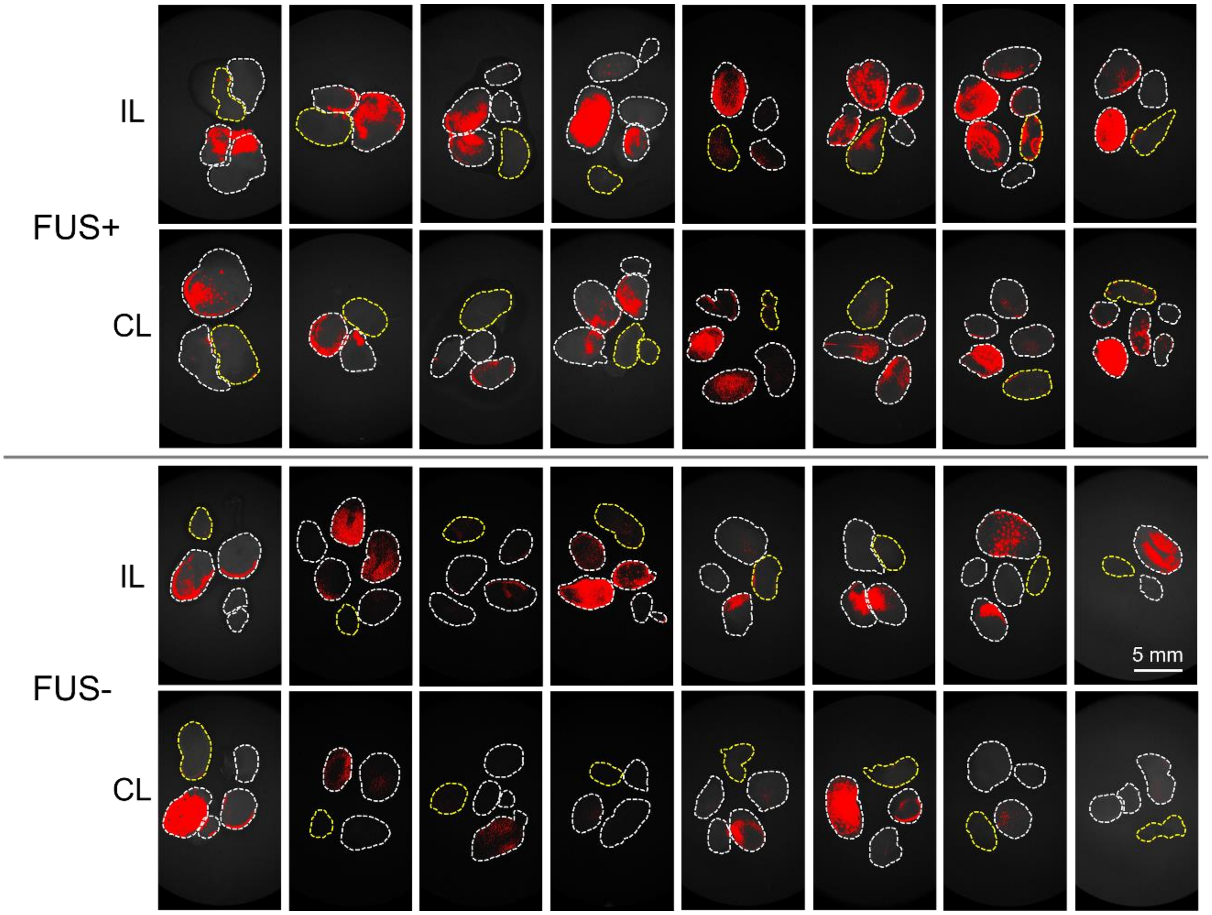

Figure S3. OA fluorescence images of cNLs from both FUS+ and FUS- conditions (n = 8 in each group). The area of OA uptake was delineated in red color. The contours of dcLN of scLN are indicated with yellow and white dotted lines, respectively.

IL: Ipsilateral to FUS/injection site, CL: Contralateral to FUS/injection site. Bar = 5 mm

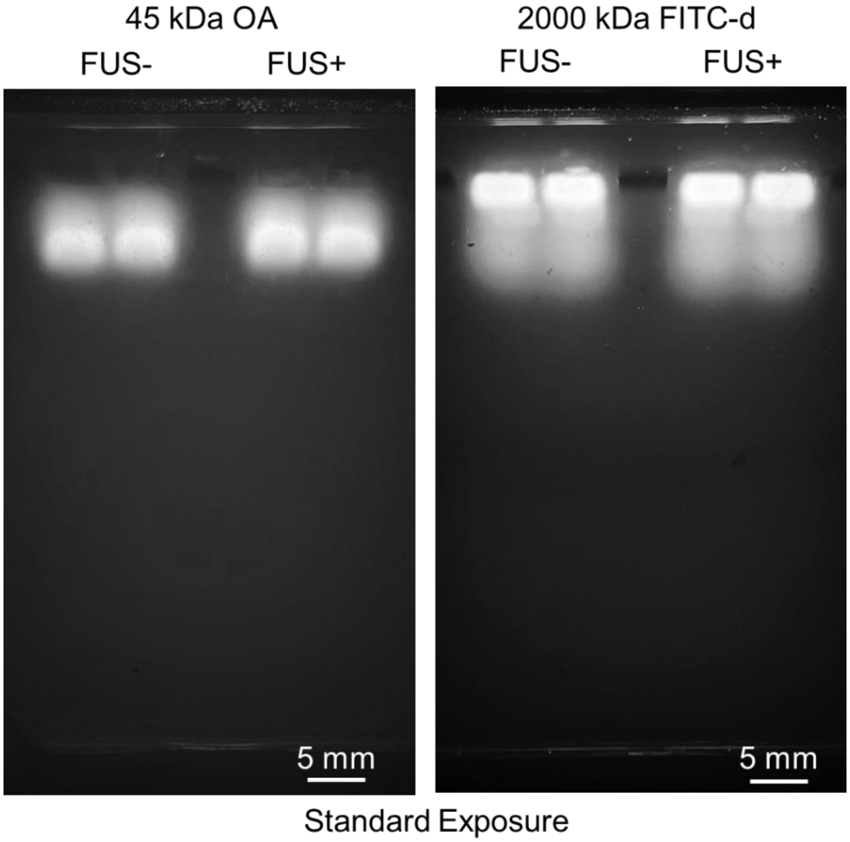

Figure S4. Full-length uncropped gel electrophoresis of Texas Red OA and FICT-d. Automatic camera exposure was used in each tracer type. Bar = 5 mm.

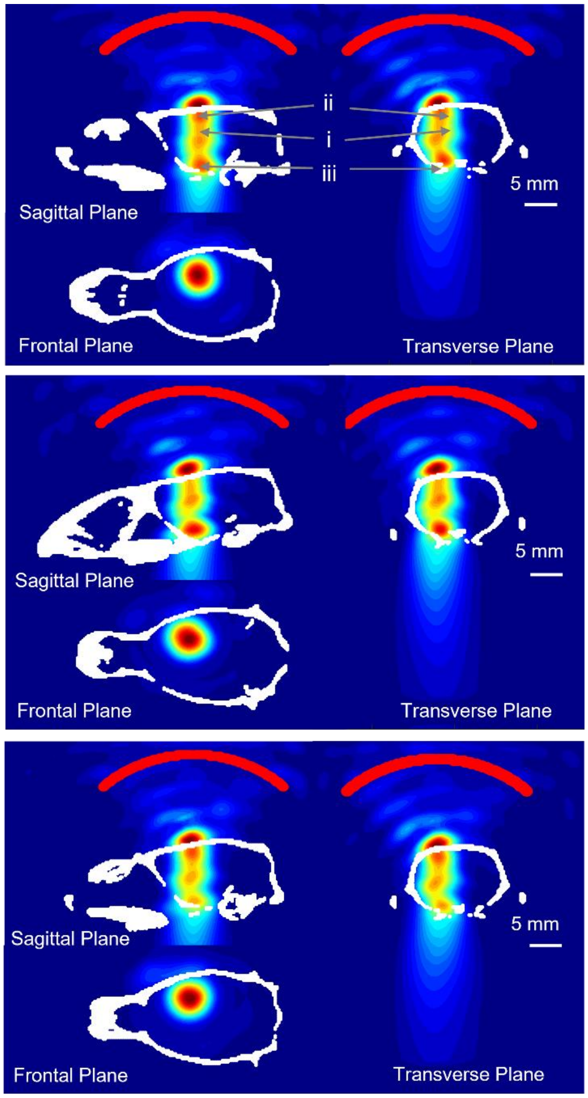

Figure S5. Numerical simulation of acoustic propagation through rat skulls (n = 3). Pseudo-colored acoustic intensity map normalized with respect to the maximum value obtained from the numerical simulation across three rat skull CT images. The profile of the transducer is marked by the red line. The sites of local pressure maxima are observed in (i) the geometric acoustic focus, (ii) the brain tissue interfacing the skull surface facing the incident sonication, and (iii) brain-skull base interface. Bar = 5 mm.

Supplementary Tables

Table S1. Measured weights, heart rates (HR), respiratory rates (RR), and SpO_2 levels of the four experimental conditions (OA and FITC-d, each with FUS (FUS+) or without sonication (FUS-)) and their group-comparison statistical results. Subscripts indicate the timing from the initiation of sonication.

Experimental Condition (n = 7 per group)	OA				FITC-d			
	**FUS+**	**FUS-**	Statistical analysis		**FUS+**	**FUS-**	Statistical analysis	
Weight (g)	286.6 ± 14.8	279.9 ± 8.3	*F* number*	*P* value	281.6 ± 12.0	281.6 ± 32.0	*F* number*	*P* value
HR/min $_{0\ min}$	223.3 ± 11.6	221.7 ± 19.5	F_t = 0.42	0.66	223.4 ± 15.5	224.9 ± 11.6	F_t = 0.67	0.52
HR/min $_{15\ min}$	213.3± 13.6	227.6 ± 29.7	F_g = 0.32	0.58	215.3 ± 13.5	226.1 ± 23.2	F_g = 0.31	0.59
HR/min $_{30\ min}$	217.3± 14.6	220.9 ± 23.5	F_i = 2.30	0.12	224.6 ± 13.6	224.3 ± 12.1	F_i = 1.42	0.26
RR/min $_{0\ min}$	60.6 ± 3.8	60.3 ± 2.9	F_t = 0.93	0.41	59.7 ± 3.5	60.6 ± 3.8	F_t = 2.88	0.08
RR/min $_{15\ min}$	59.1 ± 2.8	60.6 ± 3.0	F_g = 0.13	0.73	59.2 ± 2.5	58.6 ± 1.5	F_g = 0.08	0.78
RR/min $_{30\ min}$	59.7 ± 2.4	59.7 ± 2.1	F_i = 0.74	0.49	59.1 ± 2.5	60.1 ± 3.5	F_i = 1.30	0.29
$SpO_{2\ 0\ min}$	87.1 ± 5.3	87.9 ± 1.5	F_t = 0.94	0.40	89.1 ± 2.0	88.6 ± 2.9	F_t = 0.36	0.70
$SpO_{2\ 15\ min}$	86.9 ± 5.4	88.4 ± 3.3	F_g = 0.14	0.72	89.1 ± 3.5	89.6 ± 2.8	F_g = 0.02	0.89
$SpO_{2\ 30\ min}$	88.1 ± 5.2	88.3 ± 3.3	F_i = 0.85	0.44	89.0 ± 3.1	88.6 ± 2.9	F_i = 0.27	0.76

OA: ovalbumin tracer, FITC-d: dextran tracer. All values are given in mean ± standard deviation.
*Degrees of freedom; $F_t = F_{time}$ (2,41), $F_g = F_{group}$ (1,41), and $F_i = F_{interaction}$ (2,41)

Table S2. Weights of animals, heart rates (HR), respiratory rates (RR), and SpO_2 levels measured from the experiment that examined the OA drainage to lymph nodes, each with FUS (FUS+) or without sonication (FUS-) and their group-comparison statistical results. Subscripts indicate the timing from the initiation of sonication. Mean ± standard deviation.

Experimental Condition (n = 8 per group)	**FUS+**	**FUS-**	
Weight (g)	279.1 ± 6.4	280.0 ± 8.8	Statistical analysis
$HR/min_{0\ min}$	241.9 ± 44.3	231.1 ± 43.2	
$HR/min_{15\ min}$	236.4 ± 27.6	232.0 ± 40.5	F_{time} (4,79) = 0.29, P = 0.88
$HR/min_{30\ min}$	243.5 ± 50.4	233.9 ± 45.5	F_{group} (1,79) = 0.25, P = 0.25
$HR/min_{45\ min}$	246.9 ± 38.3	233.1 ± 34.2	$F_{interaction}$ (4,79) = 0.17, P = 0.95
$HR/min_{60\ min}$	242.4 ± 34.7	235.0 ± 34.6	
$RR/min_{0\ min}$	59.3 ± 1.0	59.6 ± 1.7	
$RR/min_{15\ min}$	58.0 ± 3.4	60.0 ± 3.5	F_{time} (4,79) = 0.69, P = 0.60
$RR/min_{30\ min}$	58.8 ± 4.5	60.0 ± 4.3	F_{group} (1,79) = 0.45, P = 0.51
RR/min_{45min}	59.0 ± 4.3	58.5 ± 3.2	$F_{interaction}$ (4,79) = 0.43, P = 0.78
RR/min_{60min}	59.8 ± 1.3	59.5 ± 3.5	
$SpO2_{0\ min}$	88.1 ± 2.4	88.3 ± 2.3	
$SpO2_{15\ min}$	88.1 ± 3.0	88.1 ± 2.2	F_{time} (4,79) = 2.22, P = 0.08
$SpO2_{30\ min}$	87.8 ± 3.7	88.5 ± 1.9	F_{group} (1,79) = 0.07, P = 0.81
$SpO2_{45\ min}$	88.8 ± 2.5	89.5 ± 2.5	$F_{interaction}$ (4,79) = 0.36, P = 0.83
$SpO2_{60\ min}$	89.1 ± 2.3	89.0 ± 3.2	

Table S3. The number of cLNs and % area showing the OA uptake from dcLN/scLN. All values are given in mean ± standard deviation (n = 8 per group).

Experimental Conditions	Side of OA injection	Number of cLNs (number of dcLN)	Area of cLNs (mm^2)	% OA uptake in dcLN	% OA updake in scLN
FUS+	IL	4.6 ± 1.1 (1.0 ± 0.0)	58.7 ± 11.9	1.4 ± 1.9	23.7 ± 5.2
	CL	4.5 ± 1.2 (1.1 ± 0.4)	54.2 ± 13.1	0.5 ± 0.6	13.3 ± 6.6
FUS-	IL	4.8 ± 1.0 (1.0 ± 0.0)	51.2 ± 12.7	0.5 ± 0.9	14.0 ± 7.7
	CL	4.4 ± 0.5 (1.0 ± 0.0)	45.9 ± 8.0	0.4 ± 0.9	10.8 ± 12.2

IL: Ipsilateral to FUS/injection site, CL: Contralateral to FUS/injection site.
